# A Strategy for Single-Run Sequencing of the Water Buffalo Genome: (II) Fast One-Step Assembly of Highly Continuous Chromosome Sequences

**DOI:** 10.3390/ani15203014

**Published:** 2025-10-17

**Authors:** Elvira Toscano, Leandra Sepe, Federica Di Maggio, Marcella Nunziato, Angelo Boccia, Elena Cimmino, Arcangelo Scialla, Francesco Salvatore, Giovanni Paolella

**Affiliations:** 1 CEINGE–Biotecnologie Avanzate “Franco Salvatore”, Via Gaetano Salvatore, 486, 80145 Napoli, Italy; toscano@ceinge.unina.it (E.T.); leandra.sepe@unina.it (L.S.); dimaggio@ceinge.unina.it (F.D.M.); nunziato@ceinge.unina.it (M.N.); elena.cimmino2@unina.it (E.C.); scialla@ceinge.unina.it (A.S.); 2 Dipartimento di Medicina Molecolare e Biotecnologie Mediche, Università Degli Studi di Napoli Federico II, Via Sergio Pansini, 5, 80131 Napoli, Italy

**Keywords:** genome assembly, water buffalo, third-generation sequencing, genome sequencing, de novo assembly

## Abstract

Genome sequencing has possibly been the greatest step in the development of advanced tools for animal genetic improvement: knowledge of gene sequences and use of haplotype markers for productivity traits can provide important improvements in yield production and optimisation of reproductive program. Next-generation and, more recently, third-generation sequencing techniques enormously increased the ability to produce sequences from single individuals and increased the interest in exome or whole-genome sequencing as an alternative to SNP chips in breeding programs as these techniques allowed for the capture of a wider range of variations, including characterisation of rare variants, structural variations, and copy number changes. Here, we present a procedure, based on fast de novo assembly and a scaffolding step, to quickly build an almost complete genome starting from long reads obtained in a single sequencing run. The procedure, applied to sequences from five water buffaloes, was able to independently build, for each individual, an almost complete high-quality genome with highly continuous chromosome sequences; in most cases, over 90% of the length of the reference chromosome was covered by less than ten long contigs. Unlike other pipelines based on slower assemblers or which require many sequencing data, in 1–2 days, the proposed procedure can go from a single run to continuous genome assembly, supporting fast analysis of large chromosome structures, potentially useful for improving animal breeding and productivity.

## Simple Summary

The development of next-generation sequencing enormously increased sequence-producing ability, opening up the opportunity to use sequences from single individuals as an alternative to SNP chips for genotyping in breeding programmes. In this context, long reads produced through the third-generation sequencing strategies are a great contribution towards genome assembly by more easily solving “difficult” regions, such as centromeric and telomeric regions, and thus increasing the interest in whole-genome re-sequencing for many livestocks. This study demonstrates that long reads from third-generation technology can be used to rapidly build, from single sequencing runs, almost complete water buffalo genomes, with highly continuous chromosome sequences.

## 1. Introduction

In recent years, the huge amount of information and technology acquired in sequencing the human genome has led to the availability of the sequence of a large number of animal genomes, including many farm breeding animals, and many genomic technologies found a role as selection tools for improving animal welfare and reproduction as well as food quality [1,2]. While at the start of the genomic era, during the 1980s, the main focus was on the development of standalone genome marker tests for inherited diseases and parentage, the availability of genome sequences became larger after the first draft of human genome in year 2000, also opening the way to the first commercial genotyping chip involving single nucleotide polymorphisms launched in 2007 [3]. The first genome sequencing of the bovine species (*Bos taurus*) was carried out on Hereford beef cattle, and provided crucial information for applications in dairy cattle selection [4]. Later, improved trait predictions were made possible by the release of complete genome sequences which granted the use of large sets of genetic markers (10,000 to 1,000,000 SNPs), tested by high-throughput genotyping platforms (DNA arrays or SNP chips) [5]. The exchange of genomic data among different countries further improved the accuracy of genomic evaluation and the prediction of the different traits of interest [6].

With the development of next-generation sequencing and subsequent reductions in sequence costs, interest in using whole-exome and genome re-sequencing as an alternative to SNP chips for genotyping in breeding programmes increased [7]. The main advantage of using re-sequencing is that it allows the capture of a wider range of variation specific to the population of interest. Moreover, the use of whole-genome sequencing (WGS) offers several additional benefits, including characterisation of rare variants, structural variations (SV), and copy number variations (CNV). The 1000 Bull Genomes Project [8] allowed the identification of several tens of millions of SNPs for various characteristics of interest and has supported a growing understanding of the genomic variation that exists both within and between populations and the functionality of different parts of the genome. Such results led to a growing interest in establishing pangenomes able to describe the genomic variation that exists within a species, an information that cannot be fully contained in a single reference sequence [9,10].

High-throughput sequencing technology has further extended genome sequencing to different species; reference genome sequences are now available for most livestock, including poultry [11,12,13], cattle [14,15], buffalo [16,17,18], yak [19,20], pig [21,22], goat [23,24], and sheep [25,26]. In addition, high-throughput sequencing technologies enormously increased the ability of producing sequences from single individuals [27], as it is becoming more usual in human diagnostics [28,29].

The present work proposes a procedure, based on fast de novo assembly and a rapid scaffolding step, which, unlike others based on slower assemblers or requiring more sequencing data, rapidly produces continuous individual genome assemblies from a single sequencing run and supports fast analysis of large chromosome structures.

## 2. Materials and Methods

### 2.1. Sequencing by Long Reads Strategy

As described in the sister article [30], five Mediterranean buffaloes, both male and female, were enrolled and for each subject, blood samples were taken in EDTA, from which genomic DNA was extracted as soon as the samples arrived at the laboratory. Genomic DNA extraction was performed following an optimised protocol to ensure a high quality and quantity of genetic material, paying attention to obtaining high molecular weight DNA, essential for the subsequent steps. DNA quality and concentration were assessed using Nanodrop (Sigma-Aldrich, St. Louis, MO, USA), Qubit 4.0 (Sigma-Aldrich, St. Louis, MO, USA), and TapeStation 4200 (Agilent Technologies, Santa Clara, CA, USA).

Whole-genome sequencing (WGS) libraries were then prepared for each sample using Oxford Nanopore’s long-read technology (Oxford Nanopore Technologies, Oxford, UK). The samples were processed with two different kits (SQK-LSK110 and SQK-LSK114), analysing the differences in terms of reads throughput. Once prepared, the libraries were loaded onto the PromethIon24 system for sequencing, lasting 80 h.

### 2.2. Read Preprocessing and Genome Assembly

The reads produced by the sequencing procedure were determined through the base-calling tool (Guppy 6.3.9, Dorado 7.1.4 and Dorado 7.2). In the base-calling procedure, reads are filtered by selecting those with an average Phred quality score larger than 10 to produce pass files to be used for further analyses. A preprocessing procedure by porechop (version 0.2.4) [31] was used to trim adapter sequences at the beginning and end of each read and to filter out chimeric reads, occasionally produced by ligation of the same adaptor to two different DNA fragments.

Shasta [32], version 0.11.1, was used for the de novo assembly steps using the configuration file Nanopore-May2022 and after filtering the input sequences on the basis of their length to discard reads shorter than 500 bases.

Ragtag v1.1.0 [33] was used for scaffolding and improving genome assembly on the basis of information present on reference genome, and -r parameter was used in order to infer gap sizes.

### 2.3. Assembly Statistics and Reference-Based Evaluation

Assembly statistics and reference-based evaluation was obtained by QUAST 5.0.5 [34,35], using the following options: *--eukaryote*, *--large*, *--min-contig 3000*, *--no-sv*. Options *--conserved-genes-finding* and *--features gene* were used to evaluate genome assembly, taking gene position into account.

Statistical analysis and plot production was conducted using R (version 4.1.0), either within the R studio environment or directly accessed from within PHP scripts as previously described [36,37,38]. Some plots were produced by taking advantage of plotly for its ability in managing dynamic plots [39,40].

Further evaluation of the assembled genome sequences was obtained by importing and analysing QUAST results within ChromoMapper (version 1.5.2), an application developed in the laboratory (manuscript in preparation). Briefly, ChromoMapper is a tool designed to comparatively evaluate different mammalian genome assemblies, starting from the results of a procedure for assembly mapping onto a reference genome such as a QUAST run. It uses information about alignment blocks, their start and end in reference and assembly coordinates, together with additional annotations to represent, at chromosomal or sub chromosomal scale, the main alignment regions, highlighting similarities and colinearity between compared sequences, points of inconsistency, discontinuities, repeated regions, and interruption in the assembled sequences. ChromoMapper starts from the alignment blocks reported within a tsv file, organises them according to the chromosome on which they map and, for each test block, calculates a set of addition parameters describing alignment block features, block end annotations, and related blocks. The output includes both tables and graphic alignment representations.

## 3. Results

### 3.1. De Novo Genome Assembly from a Single Run of Long-Read Sequencing

Long reads were produced by sequencing DNA obtained from five water buffalo individuals. The sequences, obtained by a third-generation sequencing procedure (Oxford Nanopore) described in the companion paper [30] show sequence length and quality in line with the technology (24–40× sequencing depth, 8–13 kbase average length, 11–18 kbase N50 and 14–19 mean base quality score) (Table 1). They were assembled using Shasta [32], a fast de novo assembler, as described under Methods, obtaining assembled contigs in about 1 day, a time much shorter than other assembly tools (see Section 4). The results, reported in Table 1, show that, for all the analysed individuals, contigs covering an almost complete genome could be obtained, with a total assembly length of about 2.6–2.7 Gbases, consistent with the length of water buffalo genome available in the literature [16]. For all the runs, genome assembly produced a few thousands of large contigs, starting from 14 to 40 Mbases for run 3–5, or even 50 to 60 Mbases for the first two runs, a size already comparable to full chromosome lengths. The best contig length distributions were also obtained from run1 and 2, which cover over 50% of the genome with only 70 and 54 contigs and reach 75% with 150 and 119 contigs, respectively. These numbers correspond to a N50 of 12 and 15 Mbases and a N75 of 6 and 7 Mbases.

**Figure 1:**
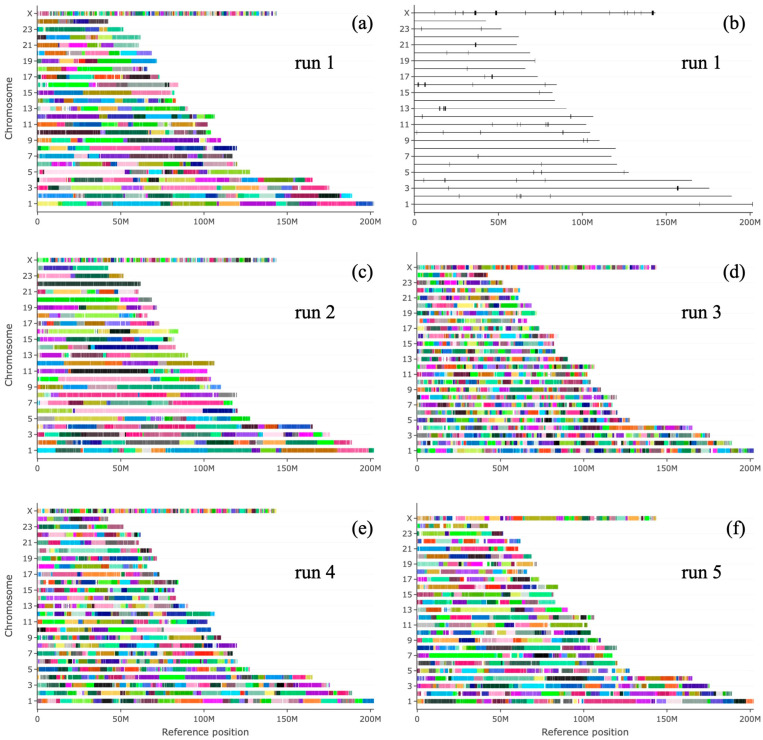
Evaluation of assembly mapping on a reference genome. (**a**,**c**–**f**) For each assembly run, contigs (coloured rectangles) are reported, aligned on the corresponding reference chromosome. (**b**) For run1, alignment gaps are represented as coloured rectangles which start at each block alignment interruption, represented as vertical black lines.

The quality of the produced contigs was evaluated by mapping each assembly onto the chromosomes of the available water buffalo genome [16] using QUAST [34,35]. In Figure 1, for each assembly run, contigs are aligned on the corresponding reference chromosome; in all cases, the reference chromosomes are covered for their whole length by only a few units or occasionally tens of very long contigs, with very small interruptions between them, as only a few tens of stretches larger than 5000 bases are missing in the alignment. Overall, the results confirm the high quality of the contigs produced after the assembly step.

**Figure 2:**
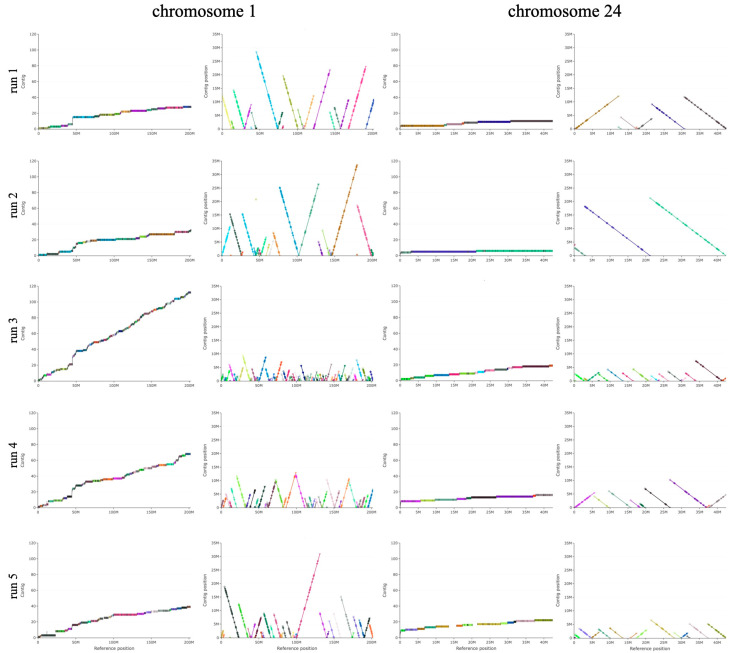
Evaluation of single chromosome coverage. For each assembly run, for chromosome 1 and 24, two different plots are reported in the first and second column. In the first one, each produced contig is reported on the *y* axis and alignment blocks are reported as coloured rectangles as a function of reference chromosome position on which they are mapped; interruptions of alignment regions are represented as vertical black lines. In the second column, a dotplot-like representation is used, where, for the same chromosomes, blocks are reported as segments tagged with start (circles) and stop (triangles); long linear stretches of segments indicate perfect correspondence between contigs and reference genome while inverted blocks are represented as segments with opposite slope.

Coverage of single chromosomes was evaluated by producing two different plots. In the first, each ordered contig is reported on a different level along the *y* axis and alignment blocks (coloured rectangles separated by vertical black lines) are positioned according to the reference chromosome on which they are mapped. In the second plot, a dotplot-like representation was used, where, for the same chromosomes, blocks are reported as segments tagged with start (circles) and stop (triangles); long linear stretches of segments indicate perfect correspondence between contigs and reference genome, while inverted blocks appear as segments with opposite sloping in the opposite direction. The results obtained for the longest and the smallest chromosome (1 and 24) are reported in Figure 2 and confirm that contigs produced by assembling the first two runs are mostly continuous, with chromosome 1 built of about 15 long contigs covering over 99% of the whole chromosomal length; the remaining 1% is strongly repetitive and corresponds to 15 small contigs all located 45 Mbases from the left end. Chromosome 24 is even more compact and is made of 3–7 long contigs.

Run 3–5 produced similar contigs, although slightly less integrated, with 40 to 120 contigs needed to cover chromosomes. In all five assemblies, highly fragmented regions were observed in the previously mentioned region located at position 45 Mbases of chromosome 1 and similarly at the start of chromosome 24; these positions are consistent with cytogenetic data with map centromeric regions in the same areas [41,42].

Table 2 reports coverage analysis for all the chromosomes from run1 assembly and shows that all of them have a global coverage of about 98–99% with a high identity percentage (about 99%). In most cases, these values are obtained by only a few tens of long contigs and a few hundred alignment blocks: L50 and L90 columns show that 50% or even 90% of chromosome length is always (L50) or mostly (L90) covered by less than ten long contigs. A notable exception is chromosome X, which shows lower coverage and higher fragmentation level, a result consistent with a known excess of heterochromatic and repeated regions, known to be more difficult to sequence and assemble [43,44,45,46]. The same or very similar results were obtained for the other four runs (Tables S1–S4); for chromosome X, a difference was observed between male and female individuals, with values of L90 of 124–228 and 42–90 contigs, respectively, possibly related to the lower sequencing depth for sex chromosomes in male individuals.

### 3.2. Reference-Driven Scaffolding Step Results in Good-Quality Genome

Genome assembly was further improved using Ragtag v1.1.0 [33] to reorder contigs into scaffolds by taking advantage of the reference genome [16] as a guide. The results, reported in Table 3, show that, for all the individuals, a scaffold set covering the complete genome could be put together by introducing a small number (1000–2000) of gaps while merging the available contigs into scaffolds. The resulting sets only use 9–10 or 16–17 scaffolds to respectively cover 50% or 75% of genome length, corresponding to an N50 of 110–117 Mbases and an N75 of 82–83 Mbases.

**Figure 3:**
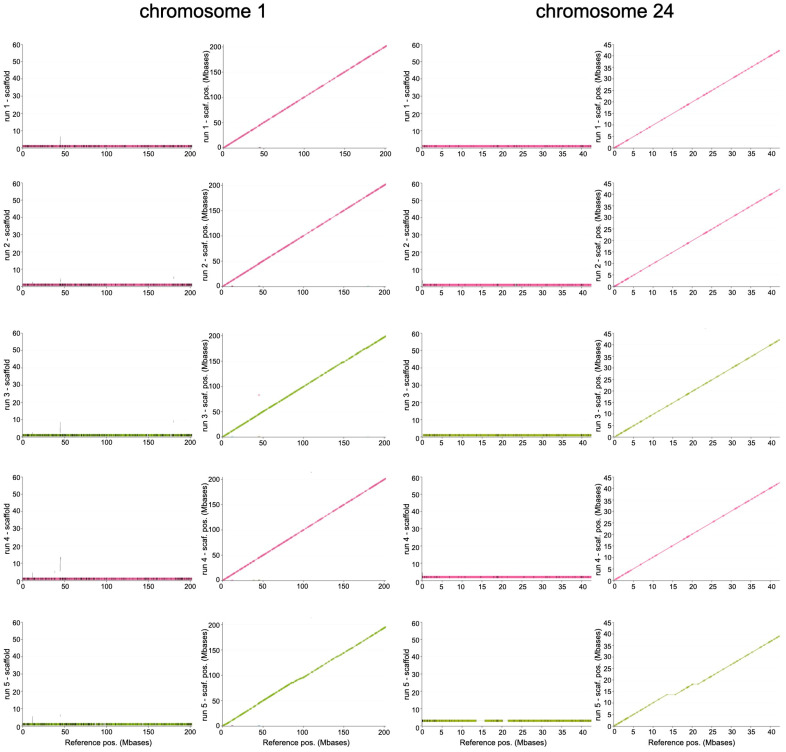
Evaluation of single scaffold chromosomes. For each run, for chromosome 1 and 24, two different plots, as in Figure 2, are reported in the first and second column. In the first one, long scaffolds are reported on the *y* axis and alignment blocks are reported as coloured rectangles as a function of reference chromosome position on which they are mapped; interruptions of alignment regions are represented as vertical black lines. In the second column, a dotplot-like representation is used, where, for the same chromosomes, blocks are reported as segments tagged with start (circles) and stop (triangles).

Chromosome coverage by the produced scaffolds was evaluated by comparing and mapping them onto the reference genome. The results show that, for all the runs, reference chromosomes are covered for their whole length by a single long scaffold, with a highly colinear alignment and a few small regions of difference (Figure S1, Table 4 and Tables S5–S8); only occasionally, larger differences are observed, such as for run5, where two larger gaps are left empty in chromosome 24 (Figure 3). In addition to the unique large scaffold, in some cases, a few smaller contigs map on the chromosome, essentially in the same areas previously indicated as corresponding to the centromeric regions (Table 4 and Tables S5–S8 and Figure 3).

## 4. Discussion

Genome sequencing has possibly been the greatest step in the development of advanced tools for animal genetic improvement in last decades; the knowledge of gene sequences and the use of haplotypes as markers for productivity traits provided important improvements in production yields and optimisation of reproductive programs [47,48,49,50,51]. The development of next-generation sequencing enormously increased the ability to quickly produce sequences, making it feasible to sequence the genome of single individuals and even to conceive the use of exome or whole-genome sequencing as an alternative to SNP chips for genotyping in breeding programmes. In addition, these techniques allow the capture of a wider range of variations present in the population of interest, including characterisation of rare variants, and structural and copy number variations, as well as analysis of methylation pattern [52]. More recently, long reads produced through third-generation sequencing strategies generated a great contribution towards producing genome sequence assemblies, which also include centromeric and telomeric regions. The production of complete genomes has, for a long time, faced the challenge of assembling “difficult” regions, such as, for example, the repeat-rich heterochromatic regions located at the centromere [53,54]. In this area, the production of much longer sequencing reads, hybrid assembly strategies [55,56], and advances in HiFi- or Hi-C-supported genome construction [57,58,59] allowed us to obtain almost complete genome sequences for many livestock species [27].

The genomes assembled within this work demonstrate that, although starting from a single sequencing run of average sequence length and quality, the use of long reads from third-generation technology can rapidly lead to an almost complete genome, with highly continuous chromosome sequences. The presented procedure, which uses a de novo assembly step followed by reference-guided scaffolding, rapidly builds high-quality genome sequences using relatively low computer resources. It is noteworthy that the assembly step already produces very long contigs showing high correspondence with the sequence water buffalo genome [16]: in most cases, over 90% of the length of the reference chromosome was covered by a small number (<10) of long contigs. The notable exception is chromosome X, which shows a higher fragmentation level, possibly related to the known higher presence of heterochromatin and repeated regions which makes the X chromosome more difficult to sequence and assemble [43,44,45,46]. In addition, chromosome X was typically less continuous in male individuals than in females, a result coherent with the fact that in males, sequences for chromosome X are only present at half dosage compared to females where X has the same dosage as autosomal chromosomes. This necessarily results in lower sequencing depth for male X chromosome, which is known to affect the quality of assembly produced by Shasta [32]. The scaffolding step finally assembled contigs in long scaffolds covering the whole length of the reference chromosomes, with a highly colinear alignment and very small regional differences. In some cases, a few smaller contigs were mapped on the chromosomes on the position of the centromeric regions, indicative of the presence of contigs with repeated sequences outside the large contigs in which the repeated region has been included.

The features of these individual genome assemblies indicate the presented strategy as a valuable option to analyse large chromosomal structures, especially considering the fact that these results may be obtained in 1–2 days, a very short time compared with similar pipelines based on other assemblers. Shasta, the assembler used in our pipeline, is known to be much faster, on average, than other genome assembly tools [32,60,61,62]. In addition, the presented results show that, when combined with third-generation sequences, assembly quality is also very good and that a relatively fast scaffolding step is more than enough to quickly produce an almost complete genome, whose level of continuity is not far from other genomes good enough to be used as a reference. Of course, the presented procedure, using a reference-guided scaffolding approach, is not fully applicable to species still lacking high-quality reference genomes. However, the problem only involves a small number of species, as nowadays for most livestock, a reference genome is available [27]. The proposed assembly pipeline based on a de novo assembly tool, compared to others using reference-based ones, has the potential to detect structural variations (SVs) and/or other genomic features. In addition, producing very long reference-independent contigs, SVs do not result in contig interruptions, and are not detected only if accidentally located in coincidence with the relatively rare contig interruptions. This makes the presented procedure a good tool, well suited to obtain, from single individuals, high-quality sequence information to be applied for animal genotyping in breeding programmes.

## 5. Conclusions

The procedure described here can produce de novo assembled genomes from a single run of third-generation sequencing. It was used to assemble single sequencing runs from five individual buffaloes, producing continuous, high-quality chromosomes obtained in a very limited time, compared with other similar procedures. The procedure uses a de novo assembly step followed by a reference-guided scaffolding one, and is strongly suited to species for which a high-quality reference genome is available. These features make the presented procedure suitable for rapidly obtaining assembled genome sequences from single individuals, an important piece of information for animal genetic improvement and genotyping in breeding programmes.

## Figures and Tables

**Table 1:** Assembly statistics for the five runs. For each assembly, number of assembled bases, length of the longest contig, N50, N75, L50, L75, and number of contigs is reported together with starting read statistics.

	Run1	Run2	Run3	Run4	Run5
sequencing depth	40×	31×	24×	34×	24×
average read length	8731.0	12,752.6	9516.5	9858.0	10,777.4
read N50	11,283	17,814	12,208	15,869	15,519
mean base quality	14.4	14.0	13.9	19.2	17.2
assembly length	2,659,831,791	2,675,885,510	2,622,221,806	2,782,932,574	2,712,441,727
longest contig	48,309,394	61,879,406	14,254,794	24,617,929	39,123,270
N50	11,819,014	14,961,077	2,831,721	5,079,353	7,100,512
N75	5,723,742	7,086,134	1,627,221	2,449,746	3,320,268
L50	70	54	290	160	119
L75	150	119	591	358	252
number of contigs	5010	4687	6724	6963	8395

**Table 2:** Statistics of assembly mapping on reference chromosomes. For run1, for the 24 autosomic and chromosome X, start and end alignment positions, coverage (%), number of mapped contigs, L90, L50, identity (%), and number of aligned blocks are reported.

	Start	End	Coverage	n Contigs	L90	L50	Identity	n Blocks
Chr 1	236	202,105,980	99.2	28	13	5	98.8	300
Chr 2	1	188,946,972	98.6	33	16	6	99.0	294
Chr 3	17,345	175,630,833	98.5	27	12	4	98.9	267
Chr 4	17	165,320,435	98.5	39	17	6	99.0	298
Chr 5	1	127,681,980	98.5	42	10	2	98.9	265
Chr 6	1	120,552,326	98.8	34	14	5	99.0	177
Chr 7	2174	117,119,118	98.9	16	9	3	98.9	173
Chr 8	28,857	119,769,169	99.0	14	8	3	98.8	184
Chr 9	1267	110,231,718	98.6	14	8	3	98.6	200
Chr 10	3243	104,521,508	98.3	16	9	2	98.8	166
Chr 11	1	102,289,349	98.2	21	12	4	99.0	154
Chr 12	718	106,433,551	98.9	15	10	3	99.1	123
Chr 13	16	90,494,031	96.5	54	13	4	98.9	202
Chr 14	1994	83,494,928	99.2	33	15	6	99.0	127
Chr 15	1	82,162,863	99.4	10	4	2	98.9	122
Chr 16	4	84,651,008	96.9	24	8	2	98.8	166
Chr 17	306	73,313,738	98.1	18	9	3	98.9	128
Chr 18	1	65,914,046	97.9	21	8	3	99.0	123
Chr 19	1	71,701,365	99.4	8	6	3	99.1	87
Chr 20	5548	68,853,047	98.8	16	7	3	99.1	110
Chr 21	1	60,856,787	98.6	9	6	3	99.0	97
Chr 22	1	62,062,344	99.7	8	6	2	99.0	98
Chr 23	20	51,730,624	98.9	17	5	2	98.8	97
Chr 24	316	42,448,106	99.4	10	5	2	99.0	75
Chr X	8	143,533,377	92.6	308	196	46	99.3	490

**Table 3:** Assembly statistics for the five scaffolded assemblies. For each scaffolded assembly, number of assembled bases, N50, N75, L50, L75, and number of gaps are reported.

	Run1	Run2	Run3	Run4	Run5
assembly length	2,666,117,353	2,683,275,268	2,636,242,927	2,788,586,393	2,717,185,276
N50	117,185,825	117,403,291	113,894,137	110,108,647	111,153,159
N75	83,353,856	83,293,950	81,597,111	82,179,143	81,805,694
L50	9	9	9	10	10
L75	16	16	16	17	17
n gaps	1068	932	2090	1492	1166

**Table 4:** Statistics of scaffolds mapped on reference chromosomes. For run1, for the 24 autosomic and chromosome X, start and end alignment positions, coverage (%), number of mapped contigs, L90, L50, identity (%), and number of aligned blocks are reported.

	Start	End	Coverage	n Contigs	L90	L50	Identity	n Blocks
Chr 1	236	202,105,980	99.2	6	1	1	98.8	296
Chr 2	1	188,946,972	98.6	4	1	1	99.0	291
Chr 3	17,345	175,630,833	98.5	6	1	1	98.9	270
Chr 4	17	165,320,435	98.6	4	1	1	99.0	294
Chr 5	1	127,681,980	98.5	12	1	1	98.9	260
Chr 6	1	120,552,326	98.9	3	1	1	99.0	167
Chr 7	2174	117,119,118	98.9	1	1	1	98.9	172
Chr 8	28,857	119,769,169	98.9	1	1	1	98.8	180
Chr 9	1267	110,231,718	98.7	1	1	1	98.6	201
Chr 10	3243	104,521,508	98.2	1	1	1	98.8	162
Chr 11	1	102,289,349	98.4	2	1	1	99.0	146
Chr 12	718	106,433,551	98.9	1	1	1	99.1	124
Chr 13	16	90,494,031	97.0	10	1	1	98.9	194
Chr 14	1994	83,494,928	99.2	8	1	1	99.0	114
Chr 15	1	82,162,863	99.4	1	1	1	98.9	118
Chr 16	4	84,651,008	96.5	2	1	1	98.8	164
Chr 17	306	73,313,738	98.1	6	1	1	98.9	122
Chr 18	1	65,914,046	97.9	7	1	1	99.0	119
Chr 19	1	71,701,365	99.4	1	1	1	99.1	82
Chr 20	5548	68,853,047	98.9	3	1	1	99.1	106
Chr 21	1	60,856,787	98.6	2	1	1	99.0	93
Chr 22	1	62,062,344	99.7	1	1	1	99.0	93
Chr 23	20	51,730,624	98.2	4	1	1	98.8	92
Chr 24	316	42,448,106	99.4	1	1	1	99.0	74
Chr X	8	143,533,377	92.7	22	1	1	99.4	491

## Data Availability

The raw data supporting the conclusions of this article will be made available by the authors on request. Some sequencing related data are currently being made available through a website on water buffalo genome and other findings of the “GENOBU” project.

## Supplementary Materials

The following supporting information can be downloaded at https://www.mdpi.com/article/10.3390/ani15203014/s1; Table S1: Statistics of run2 assembly mapping on reference chromosomes; Table S2: Statistics of run3 assembly mapping on reference chromosomes; Table S3: Statistics of run4 assembly mapping on reference chromosomes; Table S4: Statistics of run5 assembly mapping on reference chromosomes; Figure S1: Evaluation of scaffold mapping on reference genome; Table S5: Statistics of run2 scaffolds mapped on reference chromosomes; Table S6: Statistics of run3 scaffolds mapped on reference chromosomes; Table S7: Statistics of run4 scaffolds mapped on reference chromosomes; Table S8: Statistics of run5 scaffolds mapped on reference chromosomes.

## Abbreviations

The following abbreviations are used in this manuscript:

SNP	Single Nucleotide Polymorphism
WGS	Whole-Genome Sequencing
SV	Structural Variation
CNV	Copy Number Variation
