# Supplementary material for: A Strategy for Single-Run Sequencing of the Water Buffalo Genome: (II) Fast One-Step Assembly of Highly Continuous Chromosome Sequences
Source: Animals (Basel). 2025 Oct 17;15(20):3014. doi: 10.3390/ani15203014 (PMC12561557; doi:10.3390/ani15203014)
Supplement: Supplementary file 1 [file animals-15-03014-s001.zip › animals-3700261-supplementary.pdf]

Supplementary material

Table S1. Statistics of run 2 assembly mapping on reference chromosomes. For the 24 autosomic and chromosome X, start and end alignment positions, coverage (%), number of mapped contigs, L90, L50, identity (%) and number of aligned blocks are reported.

	start	end	coverage	n contigs	L90	L50	identity	n blocks
Chr 1	236	202,105,980	98.9	32	13	4	98.9	334
Chr 2	1	188,947,062	98.2	20	14	5	98.9	297
Chr 3	18,723	175,630,833	98.9	33	18	7	98.9	256
Chr 4	1	165,309,821	98.8	30	14	5	99.0	286
Chr 5	10,770	127,681,980	98.5	32	11	3	98.9	255
Chr 6	1	120,566,885	98.4	18	4	2	98.9	166
Chr 7	2,174	117,130,872	99.2	18	8	3	98.8	173
Chr 8	28,857	119,769,166	98.4	11	7	2	98.9	205
Chr 9	1,190	110,189,787	98.5	13	5	2	98.8	192
Chr 10	97,804	104,521,508	98.6	10	5	1	98.8	162
Chr 11	1	102,193,503	98.8	16	7	2	99.1	150
Chr 12	1	106,433,551	99.2	8	6	3	98.9	139
Chr 13	1	90,438,776	96.9	36	7	3	98.9	174
Chr 14	256,133	83,494,928	98.7	17	6	2	98.9	109
Chr 15	1	82,162,863	99.2	15	9	3	99.0	132
Chr 16	4	84,651,412	96.6	31	7	2	98.7	205
Chr 17	268	73,313,738	98.5	14	10	3	99.1	124
Chr 18	1	65,914,046	97.1	23	9	2	99.0	113
Chr 19	291	71,823,947	99.1	11	7	3	99.1	107
Chr 20	9,194	68,647,751	98.8	10	4	1	99.1	123
Chr 21	162,832	60,871,998	99.4	12	8	3	99.0	97
Chr 22	1	62,062,344	99.8	2	1	1	99.0	92
Chr 23	20	51,681,244	98.8	9	3	2	99.0	85
Chr 24	1	42,448,106	98.5	6	2	2	99.1	76
Chr X	8	143,533,794	91.1	306	228	42	99.3	624

Table S2. Statistics of run 3 assembly mapping on reference chromosomes. For the 24 autosomic and chromosome X, start and end alignment positions, coverage (%), number of mapped contigs, L90, L50, identity (%) and number of aligned blocks are reported.

	start	end	coverage	n contigs	L90	L50	identity	n blocks
Chr 1	142	202,105,980	97.5	112	67	20	98.9	396
Chr 2	1	188,947,000	95.6	95	64	20	99.0	385
Chr 3	49,979	175,630,833	97.2	115	67	21	99.1	358
Chr 4	1	165,309,949	97.0	94	52	15	99.0	338
Chr 5	45,993	127,681,980	98.0	97	49	16	99.1	277
Chr 6	5,718	120,450,201	96.4	82	46	15	99.0	238
Chr 7	1,781	117,219,800	95.8	77	50	15	99.0	228
Chr 8	29,032	119,769,169	98.0	72	46	15	98.9	254
Chr 9	1,562	110,231,718	96.8	64	39	11	98.9	249

Chr 10	116,899	104,493,736	96.9	62	38	11	98.9	215
Chr 11	1	102,289,349	96.8	60	29	9	99.0	205
Chr 12	22	106,433,526	98.9	76	46	17	99.1	191
Chr 13	1,789	90,491,593	96.1	74	36	13	99.0	216
Chr 14	2,010	83,494,559	98.4	54	25	9	99.1	144
Chr 15	1	82,109,037	98.6	45	26	11	99.0	170
Chr 16	4	84,650,928	93.2	62	40	10	98.8	228
Chr 17	479	73,313,738	95.9	44	30	10	99.0	154
Chr 18	5,571	65,921,361	94.6	36	23	9	99.1	127
Chr 19	291	71,700,347	98.4	43	29	10	98.9	156
Chr 20	5,548	68,701,952	95.3	31	21	7	99.0	145
Chr 21	4,512	60,855,014	99.0	31	21	8	99.1	115
Chr 22	2,884	62,062,344	95.2	38	24	8	99.0	126
Chr 23	5	51,659,662	98.1	30	17	5	98.9	103
Chr 24	206,276	42,368,580	98.8	19	12	5	99.1	83
Chr X	8	143,533,794	95.3	228	90	26	99.3	396

Table S3. Statistics of run 4 assembly mapping on reference chromosomes. For the 24 autosomic and chromosome X, start and end alignment positions, coverage (%), number of mapped contigs, L90, L50, identity (%) and number of aligned blocks are reported.

	start	end	coverage	n contigs	L90	L50	identity	n blocks
Chr 1	2,274	202,105,980	99.6	68	30	10	98.8	337
Chr 2	1	188,947,128	99.0	49	29	9	99.0	286
Chr 3	1	175,630,833	98.7	82	36	13	99.0	281
Chr 4	17	165,327,552	99.0	59	24	7	99.0	289
Chr 5	1	127,564,269	98.7	65	26	9	98.9	265
Chr 6	1	120,603,537	98.9	72	33	10	99.1	202
Chr 7	2,492	117,219,803	99.3	46	26	8	98.9	173
Chr 8	28,968	119,769,166	98.9	40	22	8	99.0	195
Chr 9	1,233	110,231,718	98.5	51	24	8	98.9	206
Chr 10	4,703	104,521,508	99.2	19	12	4	98.8	152
Chr 11	50,010	102,289,349	98.7	43	16	6	99.1	167
Chr 12	1	106,433,526	99.0	29	18	6	99.0	156
Chr 13	1	90,494,031	97.1	79	24	8	99.0	211
Chr 14	95,212	83,494,822	99.4	40	16	5	98.8	126
Chr 15	1	82,161,433	99.4	35	22	8	99.0	140
Chr 16	4	84,651,412	97.2	44	20	6	98.9	183
Chr 17	59,432	73,296,328	98.5	21	10	2	99.0	127
Chr 18	5,571	65,913,602	98.9	26	11	3	99.0	114
Chr 19	1	71,835,129	99.2	30	16	4	99.0	117
Chr 20	34,497	68,853,047	99.1	30	12	3	99.0	117
Chr 21	1	60,857,060	99.5	13	7	3	99.1	92
Chr 22	1	62,062,344	99.5	24	15	5	99.0	114
Chr 23	5	51,753,160	99.6	27	10	3	98.9	81
Chr 24	1	42,448,106	99.8	16	7	3	99.1	79
Chr X	8	143,528,151	94.9	238	124	37	99.4	426

Table S4. Statistics of run 5 assembly mapping on reference chromosomes. For the 24 autosomic and chromosome X, start and end alignment positions, coverage (%), number of mapped contigs, L90, L50, identity (%) and number of aligned blocks are reported.

	**start**	**end**	**coverage**	**n contigs**	**L90**	**L50**	**identity**	**n blocks**
Chr 1	236	202,105,980	95.6	39	23	7	98.8	300
Chr 2	1	188,947,128	96.4	42	32	10	98.9	287
Chr 3	15,825	175,630,833	97.6	51	29	8	99.0	272
Chr 4	17	165,324,538	96.4	46	25	6	98.9	277
Chr 5	1	127,681,980	93.3	55	31	9	98.9	240
Chr 6	985	120,484,419	97.7	40	17	4	99.0	173
Chr 7	1,759	117,219,800	98.0	29	16	4	98.9	169
Chr 8	28,968	119,769,166	95.7	19	13	5	98.9	170
Chr 9	1,100	110,231,718	95.8	23	14	6	98.9	202
Chr 10	3,187	104,500,905	94.8	26	19	7	98.8	170
Chr 11	1	102,289,349	96.2	22	12	4	99.1	150
Chr 12	1	106,433,551	96.9	21	15	6	99.1	127
Chr 13	64,840	90,494,031	96.7	41	9	3	98.9	184
Chr 14	2,108	83,494,559	97.4	35	12	5	98.8	115
Chr 15	1	82,129,006	99.3	20	10	4	98.9	121
Chr 16	4	84,651,412	95.9	36	12	4	98.8	175
Chr 17	401	73,313,738	96.5	18	14	5	99.0	121
Chr 18	2,617	65,920,176	96.6	33	12	4	99.1	123
Chr 19	1	71,836,124	91.2	21	17	5	99.0	105
Chr 20	19,408	68,780,587	98.7	17	9	3	99.0	108
Chr 21	4,800	60,840,679	99.7	15	7	2	99.0	95
Chr 22	1	62,062,344	88.9	18	18	4	99.0	82
Chr 23	5	51,750,430	97.7	24	7	2	98.9	86
Chr 24	4	42,448,106	91.9	22	13	5	98.9	82
Chr X	8	143,533,794	95.3	145	42	10	99.2	319

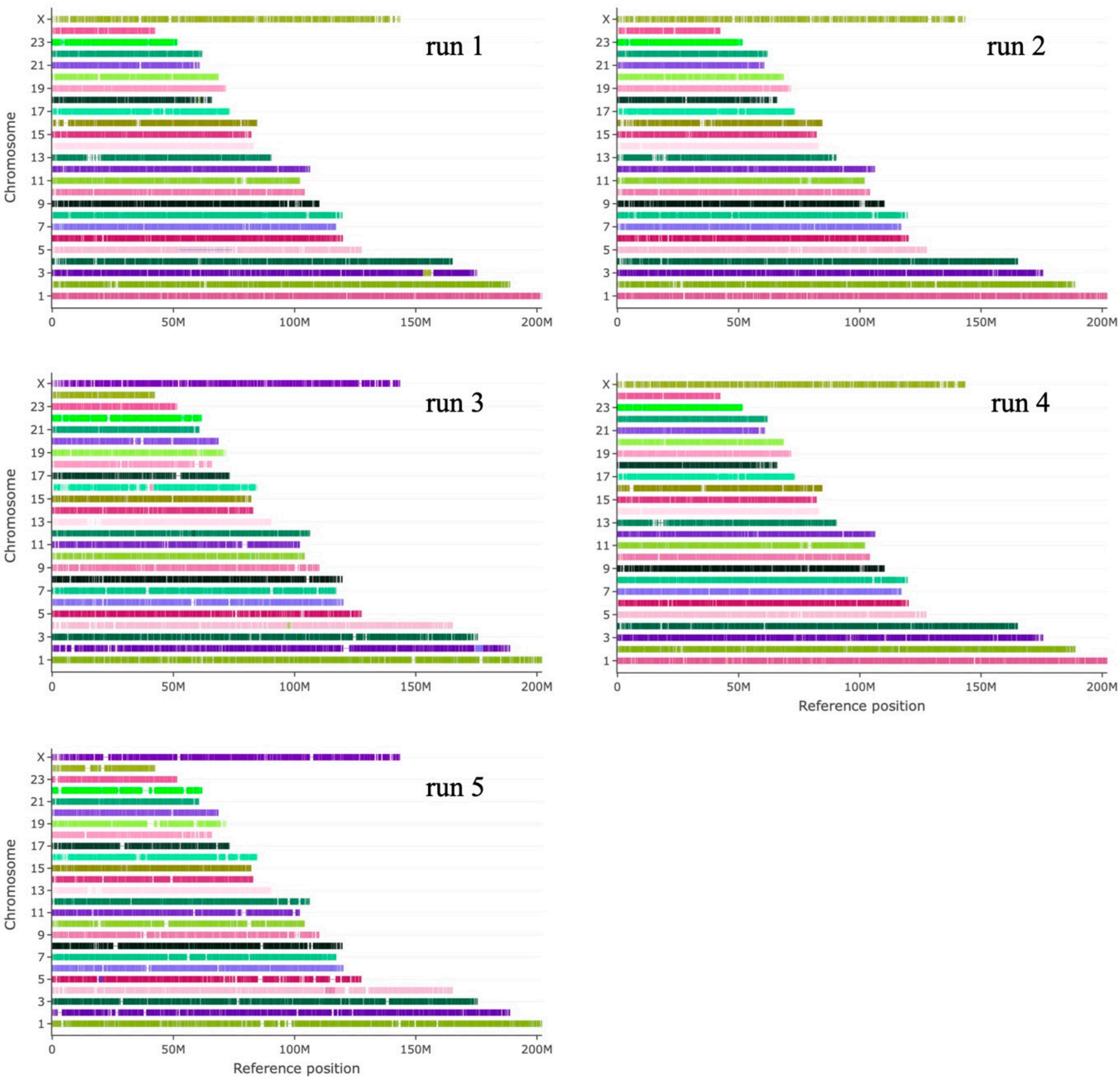

Figure S1. Evaluation of scaffold mapping on reference genome. For each run, scaffolds are reported aligned on the corresponding reference chromosome as coloured rectangles. Non aligned regions are reported as rectangle interruptions.

Table S5. Statistics of run 2 scaffolds mapped on reference chromosomes. For run 2, for the 24 autosomic and chromosome X, start and end alignment positions, coverage (%), number of mapped contigs, L90, L50, identity (%) and number of aligned blocks are reported.

	start	end	coverage	n contigs	L90	L50	identity	n blocks
Chr 1	236	202,105,980	99.0	5	1	1	98.9	328
Chr 2	1	188,947,062	98.2	2	1	1	98.9	296
Chr 3	18,723	175,630,833	98.8	2	1	1	98.9	253
Chr 4	1	165,309,821	98.8	4	1	1	99.0	280
Chr 5	10,770	127,681,980	98.4	8	1	1	98.9	250
Chr 6	1	120,566,885	98.4	4	1	1	98.9	167
Chr 7	2,174	117,130,872	99.2	3	1	1	98.8	170
Chr 8	28,857	119,769,166	98.4	1	1	1	98.9	203
Chr 9	1,190	110,189,787	98.3	2	1	1	98.8	188

Chr 10	97,804	104,521,508	98.6	1	1	1	98.9	163
Chr 11	1	102,193,503	98.8	2	1	1	99.0	144
Chr 12	1	106,433,551	99.2	1	1	1	98.9	141
Chr 13	1	90,438,776	97.0	6	1	1	98.9	172
Chr 14	256,133	83,494,928	98.8	7	1	1	98.9	108
Chr 15	1	82,162,863	99.4	1	1	1	98.9	128
Chr 16	4	84,651,412	96.7	8	1	1	98.7	209
Chr 17	268	73,313,738	98.5	2	1	1	99.0	115
Chr 18	1	65,914,046	97.2	1	1	1	99.0	108
Chr 19	291	71,823,947	99.1	1	1	1	99.0	103
Chr 20	9,194	68,647,751	98.8	2	1	1	99.2	122
Chr 21	162,832	60,871,998	99.4	1	1	1	99.0	91
Chr 22	1	62,062,344	99.8	1	1	1	99.0	93
Chr 23	20	51,681,244	98.8	3	1	1	99.0	82
Chr 24	1	42,448,106	98.5	3	1	1	99.1	73
Chr X	8	143,533,794	91.3	35	1	1	99.3	618

Table S6. Statistics of run 3 scaffolds mapped on reference chromosomes. For run 3, for the 24 autosomic and chromosome X, start and end alignment positions, coverage (%), number of mapped contigs, L90, L50, identity (%) and number of aligned blocks are reported.

	start	end	coverage	n contigs	L90	L50	identity	n blocks
Chr 1	142	202,105,980	97.4	9	1	1	98.9	382
Chr 2	1	188,947,000	95.6	6	1	1	99.0	385
Chr 3	49,979	175,630,833	97.4	4	1	1	99.1	346
Chr 4	1	165,309,949	97.1	7	1	1	99.0	341
Chr 5	45,993	127,681,980	98.4	9	1	1	99.1	271
Chr 6	5,718	120,450,201	96.4	4	1	1	99.0	223
Chr 7	1,781	117,219,800	95.8	5	1	1	98.9	221
Chr 8	29,032	119,769,169	98.1	1	1	1	98.9	243
Chr 9	1,562	110,231,718	96.8	1	1	1	98.9	239
Chr 10	116,899	104,493,717	97.0	2	1	1	98.9	208
Chr 11	1	102,289,349	96.9	2	1	1	99.1	198
Chr 12	22	106,433,526	98.9	3	1	1	99.1	177
Chr 13	1,789	90,491,593	96.1	7	1	1	99.0	209
Chr 14	2,010	83,494,559	98.4	9	1	1	99.0	137
Chr 15	1	82,162,863	98.6	2	1	1	99.0	162
Chr 16	4	84,650,928	93.1	4	1	1	98.8	220
Chr 17	479	73,313,738	95.9	1	1	1	99.0	144
Chr 18	5,571	65,921,361	94.7	3	1	1	99.1	117
Chr 19	291	71,700,347	98.4	2	1	1	98.9	150
Chr 20	5,548	68,701,952	95.3	2	1	1	99.0	140
Chr 21	4,512	60,855,014	99.2	1	1	1	99.1	104
Chr 22	2,884	62,062,344	95.2	3	1	1	99.0	122
Chr 23	5	51,669,434	98.5	2	1	1	98.9	100
Chr 24	206,276	42,368,580	98.9	1	1	1	99.1	81
Chr X	8	143,533,794	95.4	27	1	1	99.3	377

Table S7. Statistics of run 4 scaffolds mapped on reference chromosomes. For run 4, for the 24 autosomic and chromosome X, start and end alignment positions, coverage (%), number of mapped contigs, L90, L50, identity (%) and number of aligned blocks are reported.

	start	end	coverage	n contigs	L90	L50	identity	n blocks
Chr 1	2,274	202,105,980	99.6	13	1	1	98.9	305
Chr 2	1	188,947,128	99.1	3	1	1	99.0	265
Chr 3	1	175,630,833	99.0	6	1	1	99.0	244
Chr 4	17	165,337,259	99.0	4	1	1	99.0	275
Chr 5	1	127,564,269	98.7	9	1	1	98.9	236
Chr 6	1	120,603,537	99.0	5	1	1	99.1	163
Chr 7	2,492	117,219,803	99.4	4	1	1	98.9	142
Chr 8	28,968	119,769,166	98.8	5	1	1	99.0	172
Chr 9	1,233	110,231,718	98.5	5	1	1	98.9	181
Chr 10	4,703	104,521,508	99.0	1	1	1	98.9	145
Chr 11	50,010	102,289,349	98.8	5	1	1	99.1	145
Chr 12	1	106,433,526	99.3	2	1	1	99.0	138
Chr 13	1	90,494,031	97.6	8	1	1	98.9	183
Chr 14	95,212	83,494,822	99.4	12	1	1	98.8	108
Chr 15	1	82,161,433	99.6	1	1	1	99.0	116
Chr 16	4	84,651,412	97.3	6	1	1	98.9	166
Chr 17	59,432	73,296,328	98.5	3	1	1	99.0	116
Chr 18	5,571	65,913,602	98.9	7	1	1	99.0	180
Chr 19	1	71,835,129	99.2	1	1	1	99.0	98
Chr 20	34,497	68,853,047	99.1	3	1	1	99.0	104
Chr 21	1	60,857,460	99.6	2	1	1	99.1	85
Chr 22	1	62,062,344	99.9	2	1	1	99.0	98
Chr 23	5	51,753,160	99.6	5	1	1	98.9	71
Chr 24	1	42,448,106	99.9	4	1	1	99.0	70
Chr X	8	143,528,151	94.9	27	1	1	99.4	411

Table S8. Statistics of run 5 scaffolds mapped on reference chromosomes. For run 5, for the 24 autosomic and chromosome X, start and end alignment positions, coverage (%), number of mapped contigs, L90, L50, identity (%) and number of aligned blocks are reported.

	start	end	coverage	n contigs	L90	L50	identity	n blocks
Chr 1	236	202,105,980	95.9	6	1	1	98.8	307
Chr 2	1	188,947,128	96.4	1	1	1	98.9	291
Chr 3	12,737	175,630,833	97.6	2	1	1	99.0	266
Chr 4	17	165,324,538	96.5	4	1	1	98.9	278
Chr 5	1	127,681,980	93.3	7	1	1	98.9	238
Chr 6	985	120,484,419	97.7	5	1	1	99.0	175
Chr 7	1,759	117,219,800	97.9	3	1	1	98.9	171
Chr 8	28,968	119,769,166	95.7	2	1	1	98.9	172
Chr 9	1,100	110,231,718	95.8	3	1	1	98.9	208
Chr 10	3,187	104,500,905	94.8	1	1	1	98.9	168
Chr 11	1	102,289,349	96.2	1	1	1	99.1	149
Chr 12	1	106,433,551	97.3	1	1	1	99.1	132

Chr 13	64,840	90,494,031	96.7	5	1	1	98.9	178
Chr 14	2,108	83,494,559	97.5	13	1	1	98.8	107
Chr 15	1	82,129,006	99.3	1	1	1	98.9	119
Chr 16	4	84,651,412	96.2	8	1	1	98.8	178
Chr 17	401	73,313,738	96.5	2	1	1	99.0	120
Chr 18	2,617	65,920,858	96.6	7	1	1	99.1	119
Chr 19	1	71,836,124	91.2	2	1	1	99.0	103
Chr 20	19,408	68,780,587	98.6	4	1	1	99.0	108
Chr 21	4,800	60,841,519	99.7	2	1	1	99.0	90
Chr 22	1	62,062,344	88.9	1	1	1	99.0	81
Chr 23	5	51,750,430	97.8	5	1	1	98.9	84
Chr 24	4	42,448,106	91.9	3	1	1	98.9	81
Chr X	8	143,533,794	95.3	32	1	1	99.2	303
